# CD4, CD20 and PD-L1 as Markers of Recurrence in Non-Muscle-Invasive Bladder Cancer

**DOI:** 10.3390/cancers15235529

**Published:** 2023-11-22

**Authors:** Aleksandra Semeniuk-Wojtaś, Magdalena Modzelewska, Karolina Poddębniak-Strama, Sylwia Kołaczyńska, Arkadiusz Lubas, Barbara Górnicka, Anna Jakieła, Rafał Stec

**Affiliations:** 1 Oncology Department, Medical University of Warsaw, 02-091 Warsaw, Poland; 2 Pathomorphology Department, Medical University of Warsaw, 02-091 Warsaw, Poland; 3 Oncology Department, 4 Military Clinical Hospital with a Polyclinic, 53-114 Wroclaw, Poland; 4 Department of Internal Medicine, Nephrology and Dialysis, Military Institute of Medicine—National Research Institute, 04-141 Warsaw, Poland

**Keywords:** bladder cancer, NMIBC, tumor microenvironment, immune cells, CD4, CD20, PD-L1

## Abstract

**Simple Summary:**

BCG immunotherapy plays an important role in bladder cancer treatment. Unfortunately, we do not know how exactly the tumor microenvironment influences cancer cells and which cells have the most important impact on the outcome. The aim of the study was to assess how the components of the microenvironment affect tumor recurrence. We show that patients with intense CD4^+^ cell infiltration (>4.6%) or weak CD20^+^ cell infiltration (<10%), as well as patients with high PD-L1 expression on tumor cells (≥1%), could be characterized by a higher risk of recurrence. Our results provide data with potential clinical utility and may be essential for the assessment of tumor immunological status, which would be taken into account when selecting a follow-up and treatment strategy.

**Abstract:**

Introduction: A tumor microenvironment plays an important role in bladder cancer development and in treatment response. Purpose: The aim of the study was to assess how the components of the microenvironment affect tumor recurrence and to find the potential biomarkers for immunotherapy in NMIBC. Methods: The study group consisted of 55 patients with primary NMIBC. Immunohistochemistry was performed on sections of primary papillary urothelial carcinoma of the bladder. Cox proportional hazard multiple regression analysis was performed to characterize tumors with the highest probability of an unfavorable outcome. Results: Multivariate analysis confirmed that the CD4 (*p =* 0.001), CD20 (*p =* 0.008) and PD-L1 expressed on tumor cells (*p =* 0.01) were independently associated with the risk of recurrence of bladder cancer. Patients with weak CD4^+^ cell infiltration (<4.6%) and severe CD20^+^ infiltration (>10%) belong to the group with a lower risk of recurrence. The cancer in this group also frequently recurs after 12 months (*p* = 0.0005). Conclusions: The evaluation of CD4^+^ and CD20^+^ cells in the tumor microenvironment, in addition to PD-L1 on tumor cells, facilitates the determination of a group of patients with a low risk of recurrence.

## 1. Introduction

Bladder cancer (BC) is the seventh most commonly diagnosed cancer in the male population worldwide, while it drops to tenth when both genders are considered [1]. Bladder cancer incidence and mortality rates vary across countries due to differences in risk factors, detection and diagnostic practices and the availability of treatments. Approximately 75% of patients with BC present with a disease confined to the mucosa (stage Ta, CIS) or submucosa (stage T1); in younger patients (<40), this percentage is even higher [2]. Patients with Ta, T1 and CIS have a high prevalence due to long-term survival in many cases and a lower risk of cancer-specific mortality compared to T2-4 tumors [1,3].

The standard of care for patients who have high-risk non-muscle-invasive bladder cancer (NMIBC) at presentation is a combination of endoscopic resection and intravesical BCG instillation. While BCG immunotherapy is currently the best treatment for NMIBC, ~30% of patients show no response to this treatment. A study found that multifocality, lymphovascular invasion and high grade on re-TURB were independent predictors for response to BCG treatment [4]. Bacillus Calmette-Guérin intravesical treatment is associated with burdensome local and systemic side effects that could cause treatment stoppage. Serious side effects are encountered in <5% of patients [5]. In addition, BCG infections after BCG instillations have also been reported [6]. The difficulties in conducting BCG treatment are also interruptions in the availability of BCG and that can lead to the need to modify treatment, which may reduce its effectiveness. The blockade of the immune checkpoint pathway involving the programmed death 1 (PD-1) receptor and its ligands, CD274 (PD-L1) and CD273 (PD-L2), has recently been shown to be effective in the treatment of various cancers, including urothelial carcinoma (UC) [7,8,9]. Immunotherapy with checkpoint inhibitors is usually tolerable, but high frequencies of adverse events leading to discontinuation have also been reported, and thus predictive biomarkers should be established [10]. The PD-L1 expression is not enough to determine which patients may receive the largest benefit from BCG treatment; however, Zheng et al. confirmed that dysregulation of the immune microenvironment promoted the malignant progression from NMIBC to muscle-invasive bladder cancer (MIBC) and that an immune prognostic signature can stratify patients into different risk groups with distinct immunotherapeutic susceptibility, thus facilitating personalized immunotherapy [11,12].

The impact of immune components of the tumor microenvironment on the disease has been analyzed by many researchers. Galon et al. and Mlecnik and colleagues determined that tumor-infiltrating lymphocytes (TILs) affect the clinical attributes of cancer [13,14]. Thompson et al. found that patients with specimens that demonstrated high levels of lymphocyte B7-H1 expression were significantly more likely to die of RCC, even after adjusting for other pathologic features predictive of outcome [15]. Minari et al. confirmed that there were less neutrophils in a tumor at the low clinical stage than in the more advanced tumor [16]. Toge et al. and Komohara et al. proved that the presence of macrophages in the tumor was an independent factor affecting survival [17,18]. The presence of M2 macrophages in tumor cells is associated with a shorter OS. Dense infiltration of CD45R0 and CD4 cells also has a negative impact on the survival of patients [19]. Liu et al. detected that tumor-infiltrating neutrophils were independent unfavorable prognosis markers related to a high risk of recurrence in NMIBC patients and poor overall survival rates [20]. Also, a study found that high counts of Treg and TAM were associated with shorter recurrence-free survival in NMIBC patients [21].

The high efficacy of immunotherapy suggests that genomic alterations are only partly responsible for bladder cancer expansion despite the high efficacy of the fibroblast growth factor receptor pathway inhibitors [22]. It seems that the immune system and the tumor microenvironment are closely related to bladder cancer and play an important role in carcinogenesis, by maintaining and spreading the cancer, and in the treatment response. The studies conducted thus far have analyzed single, selected elements of the microenvironment, but it is not certain how exactly the tumor microenvironment (TME) influences cancer cells and which cells have the most important impact on the outcome. For this reason, a decision was made to investigate the microenvironment comprehensively. The aim of the study was to assess the components of the microenvironment in terms of the potential cells affecting tumor growth and NMIBC recurrence, as well as to find the potential biomarkers for immunotherapy. The acquired knowledge on the relationship between tumor cells and the immune system, and blood vessels, provides the basis for further research and a deeper understanding of the biology of bladder cancer, which may result in the design of novel diagnostic and therapeutic strategies. There is a high probability that the elements of the microenvironment that we decided to incorporate in this study show a significant impact on the biology of the tumor and thus prognosis. Due to the universal nature of the study’s design, the method can be successfully used in the study of other types of cancer.

## 2. Materials and Methods

### 2.1. Materials

For this retrospective study, clinical data from patients’ medical history and paraffin-embedded sections of primary papillary urothelial carcinoma from the 4th Military Clinical Hospital with Polyclinic in Wroclaw, removed during the transurethral resection of the bladder tumor (TURBT) between 2017 and 2020, were used. The study was approved by the Bioethics Committee of the Medical University of Warsaw. Patients who enrolled in the study were subjected to control cystoscopy. Patients whose follow-up period was shorter than 12 months and those with tumor recurrence within the upper urinary tract were excluded from the study. A second TURBT was performed in selected patients 2 to 4 weeks after initial resection. In those in whom neoplastic lesions were detected during the cystoscopy, TURBT was performed, and the removed tissues were transferred for histopathological examination. Histopathologically confirmed bladder cancer detected during the second TURBT was interpreted as an incomplete resection and was not assessed as a recurrence. Recurrence was defined as a reappearance of histopathologically proven urothelial carcinoma detected during control cystoscopy. The progression of the disease was defined as a deeper invasion in bladder tissue in the recurrent tumor than in the primary tumor. Time to recurrence was defined as time from the first TURBT to the first local recurrence or distant recurrence.

### 2.2. Immunohistochemistry

The tumor samples were assessed by independent pathologists before the beginning of the immunohistochemistry analysis. The hematoxylin and eosin (HE)-stained tissue blocks were retrieved and reevaluated by a pathologist, and the representative areas corresponding to tumor classification and grading were selected. The tumor zones with crush artifacts, necrosis, and regressive hyalinization were excluded. Tumor histological differentiation was graded according to the 2004/2016 WHO classification, and the assessment of the clinical stage of cancer was based on the criteria of the eight editions of tumor-node-metastasis (TNM) classification by the International Union Against Cancer (UICC, Union Internationale Contre le Cancer) [23]. Immunohistochemistry was performed on 4 μm thick tissue sections. Monoclonal mouse antibodies (DAKO) were used to evaluate the expression of the analyzed antigens (CD4—clone 4B12; CD8—clone C8/144B; CD15—clone Carb-3; CD56—clone 123C3; CD68—clone PG-M1; CD20—clone L26; CD31—clone; PD-L1—clone 28-8 pharmDX). Immunohistochemistry analyses were performed in accordance with the manufacturer’s instructions. Slides were scanned using the Hamamatsu NanoZoomer 2.0 HT. The CD31 expression was presented as a number of stained small vessels presented on 1 mm^2^ of the tumor. Counting of PD-L1^+^ cells was assessed in two steps. First, representative areas were determined using a magnification of ×10 field and then an accurate analysis was performed using a magnification of ×20 and ×40. The PD-L1 expression on tumor cells and on immune cells was assessed separately and the proportion of tumor area occupied by PD-L1 expressing tumor cells (TCs) and tumor-infiltrating immune cells (ICs) was determined. In TCs, a positive protein expression was defined as a membranous expression, and, in the case of ICs, membranous and cytoplasmatic expression were approved. The evaluation of each sample was based on the extent of PD-L1 staining, irrespective of intensity. IHC scoring was grouped by percentage and was completed on a 1–3 scale: <1%—low; 1–5%—moderate; >5%—high expression. Only intratumoral immune cells were taken into account. The intensity of the color reaction with antibodies against CD4, CD8, CD15, CD20, CD56, CD68 and CD31 was assessed using the QuPath software, version 0.2.3. The proportion of immune-cell infiltration, defined by the above-mentioned antigens, was examined in representative areas encompassing at least 1 mm^2^ of the tumor and presented as a percentage of stained cells to all cells present on the examined area. Zones with necrosis or coagulation damage were excluded from the analyses. In the cases of CD4, CD8, CD15, CD20, CD56, CD68 and CD31, IHC scoring was not grouped.

### 2.3. Statistical Analysis

All statistical analyses of the results were performed using the Statistica software, version 13.3. (StatSoft Polska SP z o. o., Cracow, Poland). If the test probability, *p*, was lower than the type I error value of 0.05, then the results were considered significant. The results are presented as frequency and percentage or as median and extreme values. The correlation analysis between the variables was calculated using the Spearman or Pearson correlation test, depending on distribution normality (Shapiro–Wilk test). To test the association between dichotomous and continuous variables, the point-biserial correlation was performed. To assess the potential influence of the examined variables derived from the immunohistochemical study on recurrence-free survival (RFS), the Cox proportional hazard multiple regression analysis was performed. The cut-off point that separates the population into two groups with a different outcome was determined using ROC analysis. RFS rates were compared using the Kaplan–Meier survival analysis, with groups stratified by determined variables, and the differences between groups were checked with the F-Cox test.

## 3. Results

### 3.1. Clinicopathological Characteristics of the Study Population

The analyzed group consisted of 70 patients. After the verification of the samples by the independent investigator, 15 patients were excluded from the study, so analyses were made for 55 patients. Detailed characteristics of the group are presented in Table 1. The adjuvant BCG treatment was administered only to 15.54% of the examined group; therefore, this feature was not included in further investigations. The overall median follow-up period was 15 (range 2–54) months. During the inspected period, neoplastic disease recurred in 31 patients, which constituted 56.36% of the investigated population. In the group of patients with recurrence of the disease, 22.5% had ≥2 recurrences of the disease, 9.6% had diagnosed muscle-invasive bladder cancer (MIBC) during the first recurrence and 19.3% had a cystectomy due to bladder cancer (MIBC or high-risk NMIBC). The median time to recurrence was 9 (range 2–40) months.

### 3.2. Immunohistochemical Assessment of the Tumor Microenvironment Components

**Scheme 1:**
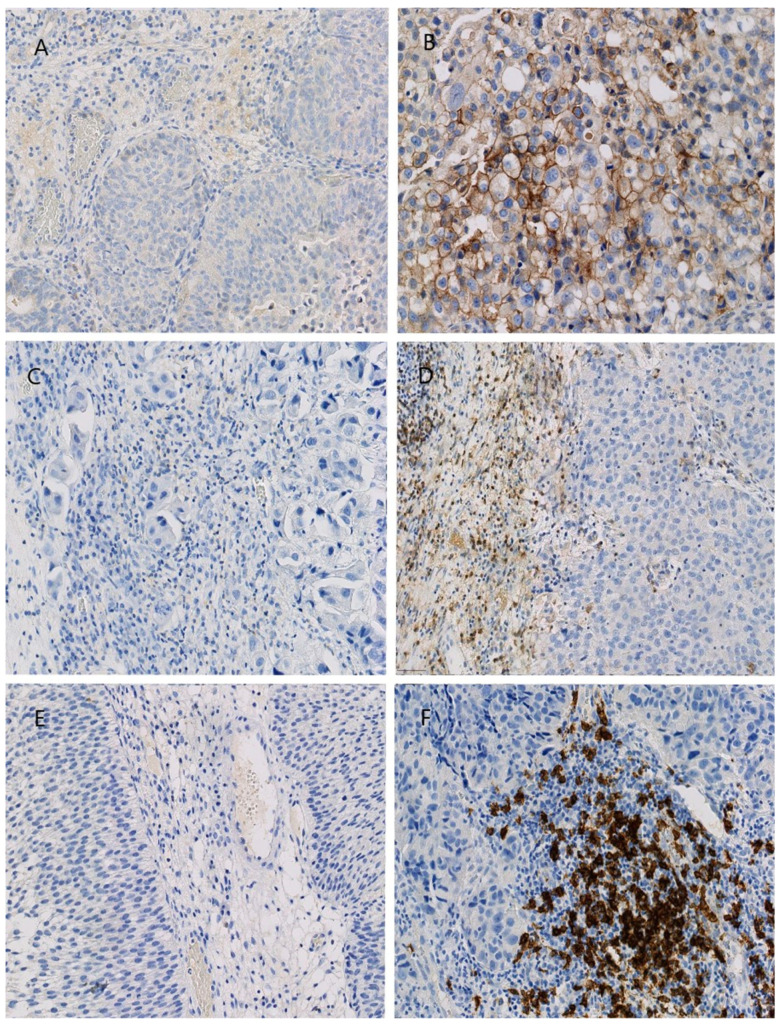
Immunohistochemical stains presenting high-grade tumors (×20): (**A**) low PD-L1 expression; (**B**) high PD-L1 expression; (**C**) low CD4^+^ density; (**D**) high CD4^+^ density; (**E**) low CD20^+^ density; (**F**) high CD20^+^ density.

The levels of CD4, CD8, CD15, CD56, CD68, CD20, CD31 and PD-L1 in primary papillary bladder cancer removed during transurethral resection of the tumor were evaluated using IHC. Expression of the analyzed protein was confirmed in 50 of the examined tissues (90%). The expression of PD-L1 on tumor cells was determined as low in 42 patients (84%), moderate in 5 patients and high in 3 patients (10% and 6%, respectively). The expression of PD-L1 on immune cells was low in 17 patients (34%) and moderate also in 17 patients (34%). High expression was found in 16 patients (32%). The details of these results are summarized in Table 2. Representative photographs are presented (Scheme 1).

### 3.3. Correlations between the Microenvironment Components

In the analyzed tumor samples, a correlation between examined TME components was found. Detailed data about the analyzed variables that are statistically significant are presented in Table 3. No statistically significant correlation was found between PD-L1 expression on tumor cells and the remaining components of the microenvironment.

### 3.4. Correlations between the Microenvironment Components and Clinical and Pathological Variables

Correlations of CD4, CD8, CD15, CD20, CD56, CD68, CD31 and PD-L1 on the expression of tumor and immune cells in non-muscle-invasive bladder cancer with patient age, histopathological malignancy (LG or HG tumors), the depth of bladder-wall invasion by the tumor and the diameter and number of tumor lesions were assessed. We also evaluated the correlations between the TME components and the recurrence, progression and cystectomy, as well as the time to tumor recurrence, progression and cystectomy.

The analyses reveal a correlation between CD4, CD15 and CD68 and between the histopathological malignancy of the NMIBC (r = 0.4832, 0.3343 and 0.3729, respectively; *p* = 0.000, 0.018 and 0.008, respectively). CD15 correlated with the recurrence (r = 0.2990, *p* = 0. 035) and CD20 with the NMIBC in the recurrent tumor (r = −0.4024, *p* = 0. 034). The frequency of CD68 correlated with the depth of bladder-wall invasion by the cancer (r = 0.4167, *p* = 0.002) and with the time to cystectomy (r = 0.8857, *p* = 0. 018). Also, PD-L1 expression on tumor cells correlated with the time to cystectomy (r = 0.8366, *p* = 0.037). PD-L1 expression on tumor cells additionally correlated with the presence of many recurrences (r = 0.4065, *p* = 0.032). When analyzing the expression of the PD-L1 protein on immune cells, a statistically significant relationship was found between patient age (r = 0.2989, *p* = 0.034) and the depth of the bladder-wall invasion by the cancer (r = 0.3139, *p* = 0.026).

In addition, we found a correlation trend between the PD-L1 protein on tumor cells, as well as CD15^+^ cell density, and the depth of the bladder-wall invasion by the cancer. There was also a correlation between CD8^+^ cell density and the age and presence of many recurrences, but it was not statistically significant.

### 3.5. Evaluation of the Prognostic Value of the Tumor Microenvironment in Non-Muscle-Invasive Bladder Cancer

The CD4, CD20 and PD-L1 expressed on tumor cells were independently associated with the risk of recurrence of bladder cancer in the Cox proportional hazard multiple regression analysis of recurrence-free survival (Table 4).

**Figure 1:**
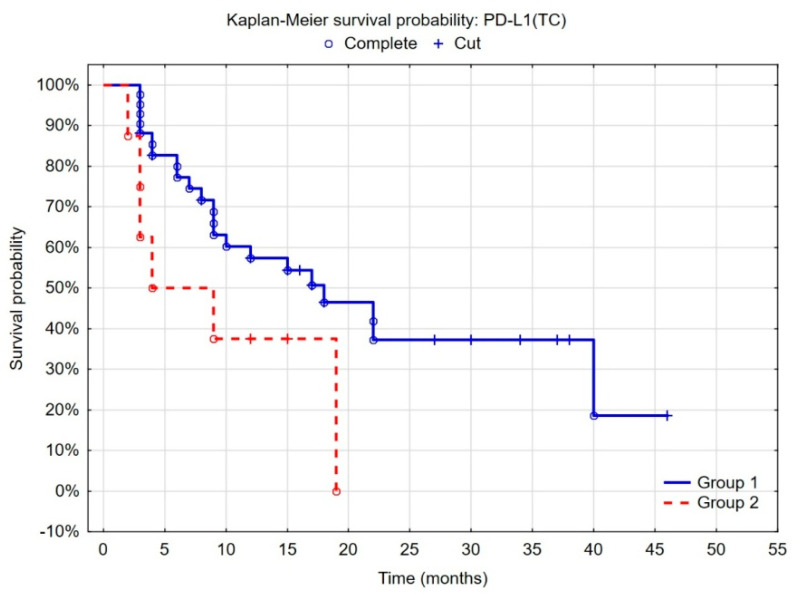
Kaplan–Meier curves of recurrence-free survival by risk groups, according to PD-L1 expression on tumor cells. (Group 1 = low expression, <1% of tumor cells; group 2 = moderate and high expression, >1% of tumor cells); F-Cox test: *p* = 0.07601.

In the case of PD-L1, analyses revealed that the probability of tumor recurrence was similar in patients with high and moderate PD-L1 expression on tumor cells. The significant decrease in the risk of recurrence was found only in patients with low PD-L1 expression (<1%) on tumor cells compared to the others (Figure 1).

**Figure 2:**
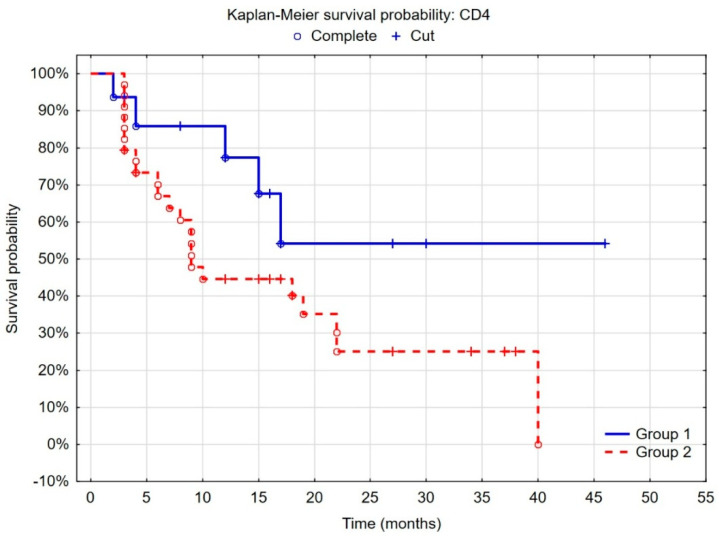
Kaplan–Meier curves of recurrence-free survival by risk groups according to CD4^+^ cell density determined by ROC curves. (Group 1 = low CD4 expression (<4.6%)); F-Cox test: *p* = 0.00593.

**Figure 3:**
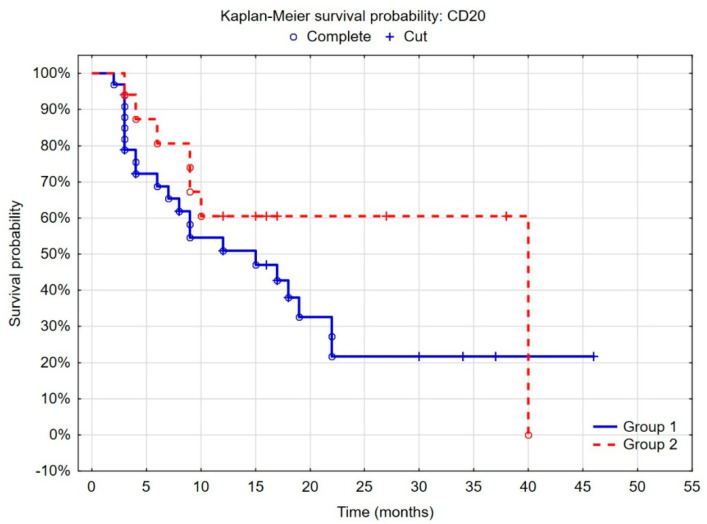
Kaplan–Meier curves of recurrence-free survival by risk groups according to CD20^+^ cell density determined by ROC curves (Group 1 = low CD20 expression (<10%)); F-Cox test: *p* = 0.05215.

When patients were stratified into groups according to the cut-off points determined by the ROC curves and considering the best recurrence predictive value, we found that the probability of recurrence was lower in the group of patients with low CD4^+^ density (<4.6%), defined by statistical analyses, than with high CD4^+^ density (*p* = 0.005). Regarding the CD20^+^ cells, the separate point determined by the same statistical method was 10%, and patients with high CD20^+^ density (>10%) had a lower risk of recurrence (*p* = 0.052) (Figure 2 and Figure 3).

**Figure 4:**
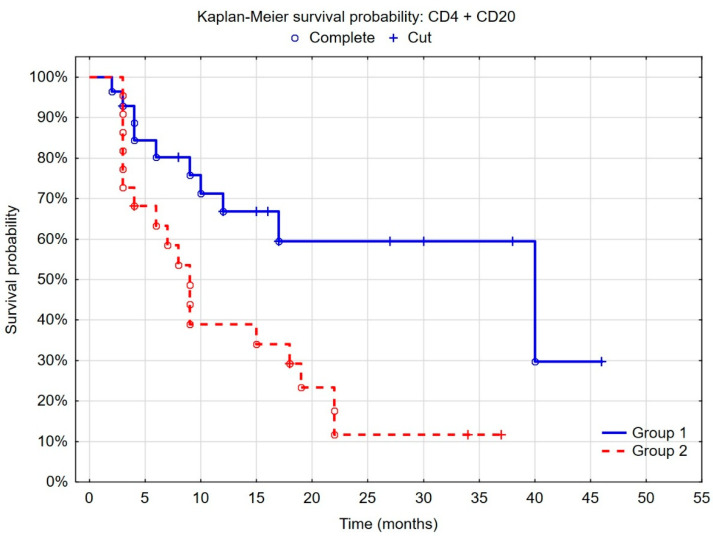
Kaplan–Meier curves of recurrence-free survival by risk groups. (Group 1 = low CD4^+^ density according to ROC (<4.6%) and high CD20^+^ density according to ROC (>10%); group 2 = high CD4^+^ density (>4.6%) according to ROC or low CD20^+^ density (<10%) according to ROC or PD-L1 expression on tumor cells (≥1%)); F-Cox test: *p* = 0.00056.

As a tumor microenvironment is a dynamic space and cells interact with each other, we decided to separate the group of patients with the highest risk of an unfavorable outcome. We found that patients with weak CD4^+^ cell infiltration and severe CD20^+^ cell infiltration belong to the group with a low risk of recurrence and the cancer in this group rarely occurs after 10 months (*p* = 0.0005) (Figure 4). Patients with intense CD4^+^ cell infiltration or weak CD20^+^ cell infiltration, as well as patients with high PD-L1 expression on tumor cells (≥1%), could be characterized by a higher risk of recurrence. In both groups, the second tumor was usually diagnosed during the first year after the primary tumor resection, but, in the first group, the probability of recurrence was significantly lower.

## 4. Discussion

In the present study, we examined the prognostic value of the tumor microenvironment in NMIBC patients. We demonstrated a correlation between CD4^+^ and CD20^+^ tumor-infiltrated lymphocytes (TILs) in the primary tumor and clinical outcomes. Moreover, we identified that patients with a high risk of recurrence could be characterized by severe CD4^+^ and weak CD20^+^ cell infiltration. In addition, moderate and high PD-L1 expression (≥1%) on tumor cells might be recognized as a marker of unfavorable prognosis, but this cannot be analyzed as a single factor.

The connection between the density and composition of immune infiltration and the outcome have been analyzed, but the studies published thus far have presented inconsistent results. In many publications, it has been found that abundant CD3^+^ and CD8^+^ infiltration correlates with a longer survival without cancer in patients with NMIBC [10,19]. The CD3 antigen is connected with the T-cell receptor (TCR) complex and participates in the recognition of antigens and subsequent activation of immunocompetent T lymphocytes, while CD8^+^ T cells are called cytotoxic T cells and are able to kill malignant cells after activation by the secretion of cytokines, the release of cytotoxic granules along the immune synapse and caspase cascade activation [24,25].

In our study, we found that CD4^+^ T cells (known also as T-helper cells), similarly to CD20^+^ cells (B cells), were the main type of lymphocytes influencing the outcome. Similar data were published by Zhang et al., who discovered that CD4^+^ T cells belong to the cells that are significantly more frequent in the high-risk group with a poorer prognosis [26,27]. The essential function of CD4^+^ T cells was further proved by Oh et al., as they detected that gene signatures of proliferating cytotoxic CD4^+^ T cells are associated with a response to a PD-1 blockade in bladder transitional cell carcinoma patients [28]. Unfortunately, our results are contradictory to some published data. Karpina et al. did not find a significant difference in infiltration by CD4^+^ or CD20^+^ between recurrent and non-recurrent NMIBC patients, and they detected lower numbers of infiltrating CD3^+^ and CD8^+^ T cells in the group of patients without recurrence. Other studies reported that high CD4^+^ T-cell density in the TME is associated with a low risk of recurrence [29,30]. It is difficult to determine the source of the differences between the studies, but there were dissimilar characteristics of the analyzed populations. In the Karpina et al. study, patients were included with papillary urothelial neoplasm of low malignant potential (PUNLMP) and low-grade NMIBC, and, in our study, we analyzed patients with low-grade and high-grade NMIBC. Furthermore, in the Karpina et al. study, patients with multiple tumors and patients with a solid or flat aspect of the tumor were excluded. Moreover, in the Karpina et al. study, the mean recurrence time in recurrent cases was longer than in our study. Despite this, the recurrence rate was similar in both studies. On the other hand, there are also divergences between the end points of the studies. Karpina et al. found that the cells correlated with cancer recurrence. We separated a group of patients with a high risk of recurrence also after 12 months after the primary tumor resection. What is more, CD4^+^ T cells can be split into four lineages, encompassing Th1, Th2, Th17 and Tregs, that have an ambivalent role in cancer. CD4^+^ T cells subsets are plastic and can be converted to another lineage during tumor growth characterized by the opposite function [27], so an antitumor response depends on the cytokines presented in the tumor microenvironment and may be changed. Nevertheless, CD4^+^ T cells are crucial for destroying cancer. Investigators have demonstrated that CD4^+^ T cells are essential for the effective induction and maintenance of cytotoxic CD8^+^ cells through both promoting the production of inflammatory cytokines and the direct licensing of dendritic cells [31]. The CD4^+^ Th1 subtype is also responsible for DC licensing, which is necessary for DC maturation [32,33]. Additionally, the activity of CD4^+^ Th1 cells influences tumor-killing promotion by NK and M1-type macrophages [34,35]. As a consequence, CD8^+^ T cells maturing in the absence of CD4^+^ T cells fail to fully activate cytotoxic abilities leading to the expansion of anergic CD8^+^ T cells with dysfunctional phenotypes [36,37,38]. Additionally, CD4^+^ T cells have the ability to induce tumor rejection via the major histocompatibility complex class II (MHC II)-dependent pathway as well as regulation by Tregs.

In the case of CD20^+^ cells, our findings were similar to previous publications; although, in another investigation no significant difference in infiltration by CD20^+^ cells between recurrent and non-recurrent NMIBC patients was found [28]. Researchers have reported that CD20^+^ cells were significantly more frequent in MIBC than in high-grade NMIBC, and we detected that low CD20 expression in accordance with high CD4 expression is a marker of unfavorable outcomes [27]. It has been found that tumor-infiltrating B cells could promote bladder cancer development, and it might seem that mechanisms involved in this process are also implicated for recurrence [39]. It has also been reported that the increased presence of CD20^+^ follicle-like structures (FLSs) in tumor tissue may be associated with increased survival in muscle-invasive bladder cancer patients. It seems that a favorable outcome is connected with FLSs as investigators have also found some cases of FLSs that were absent despite the presence of CD20^+^ cells in the tissues, and, in the second group, cancer-specific survival was shorter [40]. Previously, it has been reported that tertiary lymphoid structures (TLSs) correlated with favorable prognosis for the patients [41], and it appears that Zirakzadeh et al. detected that B cells are also able to organize in FLSs in patients with improved prognosis. Moreover, it has been speculated that the degree of TLS formation and TLSs maturity may be associated with the aggressiveness of the disease and the severity of the stromal inflammatory response [42]. B cells are known as producers of immunoglobulins that bind to pathogens. However, they also have the ability to uptake and draw up circulating antigens, with the purpose to present them in the MHC class II for CD4^+^ T cells [43], so B cells may act as antigen-presenting cells, leading to the activation of T cells.

PD-L1 is expressed by cancer cells as well as some immune cells. Recently published studies have shown that 4.6–46% of NMIBC cases express PD-L1, whereas in our study the expression was detected in 6% of examined tissues [44,45,46,47]. Similar to other reports, PD-L1 expression on infiltrating immune cells was higher than on cancer cells. In addition, we found that PD-L1 expression on tumor cells ≥1% is connected with an unfavorable outcome associated with a higher probability of cancer recurrence after the first year following primary tumor resection. Similar results regarding RFS depending on PD-L1 expression have been published [27,48]. Other reports have presented opposite data [49,50,51,52], but, as we detected, the PD-L1 expression cannot be analyzed separately as a biomarker because the immune component of the tumor is a dynamic space.

In this situation, the expression of a single antigen is not enough to determine the outcome. The probability for the appropriate determination of patient risk is higher when we assess a few elements of the microenvironment. The analysis of CD4^+^ and CD20^+^ tumor-infiltrated lymphocytes as well as PD-L1 expression on tumor cells make it possible to determine a group of patients with a high risk of recurrence. In addition, in this group, the second cancer could also be recognized after 12 months following primary tumor resection, which means that this group of patients should be closely monitored with cystoscopy for a few years after the primary TURBT. It is very important mainly when we take into consideration that almost 10% of patients had diagnosed muscle-invasive bladder cancer (MIBC) during the first recurrence. The unfavorable outcome in this group of NMIBC patients might be associated with TIL dysfunction caused by the lack of CD4^+^ cells in the tumor microenvironment, which play a key role in antitumor immunity [53]. This small frequency could be connected with some kind of poorly immunogenic cancer cell variants, while in the same tissue samples an immune infiltration was generally not large and only an abundance of CD20^+^ cell infiltration was found. In this group of patients, a rich CD20^+^ cell infiltration cannot be recognized as enough to secure tissues for cancer recurrence because the CD4^+^ cells are dysfunctional and are not able to induce the proper cytotoxicity of other cells. They need the support of anti-PD-L1/PD-1 treatment. What is more, it has been detected that in melanoma patients a high CD20^+^ expression was connected with significant benefits caused by treatment with PD-L1/PD-1 pathway inhibition [54,55], so we can presume that in NMIBC patients this would be the same. There are also data indicating that CD4 cells are a marker of response [28,56,57], but it seems that the baseline level is less important than their ability to increase during treatment [58]. In this situation, we can assume that patients in the second group characterized by high CD4^+^ cell infiltration will benefit from anti-PD-L1/PD-1 treatment as well. However, we have detected that CD4^+^ expression correlates with PD-L1 expression on immune cells, and this could be a sign of T-cell exhaustion [59]. It seems that exhausted T cells are not the aim of anti-PD-L1/PD-1 treatment [53].

Overall, we concentrated on finding a marker of recurrence in the immune component of the tumor that reflects tumor aberrations, so we did not include classical risk factors in the analysis, which are used to determine the risk of tumor progression to muscle-invasive status, such as tumor stage, tumor grade or number of tumors. Thanks to that, we found potential biomarkers of immunotherapy, as using PD-L1 expression is not enough to determine the effectiveness of immunotherapy [60,61]. Moreover, the discussed markers are simple, feasible and relatively inexpensive.

This study has the known limitations of retrospective design. In addition, the number of cases remains comparatively small, and thus the results need further validation, preferably in prospective studies. In addition, in the study, patients were included who underwent TURBT despite their adjuvant BCG treatment status and that might have had an impact on the outcome.

## 5. Conclusions

The infiltration of both the T and B cells as well as PD-L1 expression on tumor cells have emerged as important players in the immune response against cancer. This observation has potential clinical utility and may be essential for the assessment of tumor immunological status, which, in future, would be taken into account when selecting a follow-up and treatment strategy. The data from our study have enhanced the knowledge about biomarkers in patients with non-muscle-invasive bladder cancer and indicate a course of additional analyses. However, further studies aimed at better understanding the changing tumor immunogenicity during bladder cancer development are warranted. In addition, as it is a retrospective study, the results need to be validated with a prospective study.

## Figures and Tables

**Table 1:** Patient characteristics.

Feature	Number of Patients (*n*)	Percentage (%)
Stage		
Tis	1	1.8
Ta	36	65.5
T1	18	32.7
Concomitant Tis		
Yes	1	1.8
No	54	98.2
Grade		
LG	26	47.3
HG	29	52.7
Number of tumors		
1	31	56.4
2–3	18	32.7
≥4	5	9.1
Not known	1	1.8
Diameter of the largest tumor		
<1 cm	4	7.3
1–2.5 cm	26	47.3
≥3 cm	21	38.2
Not known	4	7.3
BCG adjuvant treatment		
Yes	8	14.5
No	47	85.5

Tis—carcinoma in situ (CIS), “flat tumor”; Ta—non-invasive papillary carcinoma, exophytic tumors confined largely to the mucosa; T1—tumor invades subepithelial connective tissue; LG—low-grade tumors; HG—high-grade tumors.

**Table 2:** Immunohistochemical staining for CD4, CD8, CD15, CD20, CD56, CD68 and CD31 in all analyzed samples.

Feature	Median	Minimum	Maximum
CD4 [%]	7.19	0.07	24.41
CD8 [%]	9.53	1.80	31.86
CD15 [%]	4.32	0.77	18.92
CD20 [%]	4.07	0.20	27.76
CD56 [%]	0.54	0.05	8.21
CD68 [%]	22.19	10.14	44.06
CD31^+^ vessels (on 1 mm^2^)	83.00	23.00	244.00

**Table 3:** Statistically significant correlations in the immune component of the tumor microenvironment in all analyzed samples.

	R	*p*
CD4 [%] & CD15 [%]	0.463	<0.001
CD4 [%] & CD20 [%]	0.376	0.007
CD4 [%] & CD31 (na 1 mm^2^)	−0.328	0.019
CD4 [%] & PD-L1 (IC): 1 = low; 2 = moderate; 3 = high	0.403	0.003
CD8 [%] & CD20 [%]	0.35	0.012
CD8 [%] & CD56 [%]	−0.3	0.034
CD8 [%] & CD68 [%]	0.366	0.008
CD56 [%] & CD68 [%]	−0.316	0.024

R—correlation coefficient; *p*—statistical significance level.

**Table 4:** Multiple analysis of relationship between recurrence-free survival and selected pathological variables in all analyzed samples.

	HR	95% Confidence Interval	*p*
CD4 [%]	1.19	1.07–1.32	0.001
CD8 [%]	1.00	0.90–1.12	0.87
CD15 [%]	1.06	0.93–1.20	0.33
CD20 [%]	0.90	0.84–0.97	0.008
CD56 [%]	0.83	0.62–1.10	0.2
CD68 [%]	0.97	0.89–1.04	0.44
CD31^+^ vessels (on 1 mm^2^)	1.004	0.99–1.01	0.34
PD-L1 (TC) low vs. high	0.05	0.008–0.29	0.01
PD-L1 (TC) moderate vs. high	0.05	0.003–0.80	0.19
PD-L1 (IC) low vs. high	1.6	0.51–4.98	0.14
PD-L1 (IC) moderate vs. high	0.58	0.18–1.87	0.13

HR—hazard ratio; *p*—statistical significance level.

## Data Availability

Data are contained within the article.
